# Supplementary material for: Measuring liquidity in Indian stock market: A dimensional perspective
Source: PLoS One. 2020 Sep 4;15(9):e0238718. doi: 10.1371/journal.pone.0238718 (PMC7473572; doi:10.1371/journal.pone.0238718)
Supplement: S1 Fig — Displays the impulse response functions of liquidity measures across the Quintile groups over an observation period of 10 days. (DOCX) [file pone.0238718.s001.docx]

S1 Fig. Impulse response functions of liquidity measures across the quintile groups.

**S1 Fig 1. Impulse Response Function for 1st Quintile**

**S1 Fig 2. Impulse Response Function for 2nd Quintile**

**S1 Fig 3. Impulse Response Function for 3rd Quintile**

**S1 Fig 4. Impulse Response Function for 4th Quintile**

**S1 Fig 5. Impulse Response Function for 5th Quintile**
