# Supplementary material for: Measuring liquidity in Indian stock market: A dimensional perspective
Source: PLoS One. 2020 Sep 4;15(9):e0238718. doi: 10.1371/journal.pone.0238718 (PMC7473572; doi:10.1371/journal.pone.0238718)
Supplement: Attachment — Submitted filename: Response to Reviewers.docx [file pone.0238718.s002.docx]

**Measuring liquidity in Indian stock market: A dimensional perspective**

| **Comments** | **Response** |
| --- | --- |
| Reviewer #1 |  |
| 1. For readers to quickly catch the contribution in this work, it would be better to highlight major difficulties and challenges, and your original achievements to overcome them, in a clearer way in abstract and introduction. | This study eliminates the ambiguities related to market liquidity by precisely measuring it by using popular and proven liquidity measures. (Included in the manuscript on page no. 2 in Abstract)  Notably, a major challenge while undertaking this study was the selection of appropriate liquidity measures across the four dimensions since the previous literature propounds numerous liquidity measures and their variants. This was resolved by selecting widely acknowledged liquidity measures through extensive analysis of a pool of literary works published on market liquidity. (Included in the manuscript on page no. 4 in Introduction in paragraph 1) |
| 2. The authors must explicitly state the novel contribution of this work, the similarities and the differences of this work with their previous publications. | Moreover, this study offers a novel approach of inclusively studying market liquidity by virtue of widely accepted liquidity measures in an order-driven market system. Further, estimating interdependencies between liquidity dimensions by using daily frequency data and emphasizing the patterns formed thereon is a distinct attempt made with reference to an equity market and will enable traders to effectively optimize their trading strategies. (Included in the manuscript on page no. 5 in Introduction in paragraph 1)  Similarities and the differences of this work with previous publications are included in the manuscript in Conclusion on page no. 24 in paragraph 2 and on page no. 25 in paragraph 1. |
| 3. The discussion section in the present form should be strengthened with more details and justifications. | Included in the manuscript from page no. 13 to 24 in Results and Discussion |
|  |  |
| Reviewer # 2 |  |
| 1. I would suggest to modify the introduction by adding a brief explanation of the concepts introduced in the abstract such as: depth, breadth, tightness, and so on. | Additionally, Kyle [5] mentions that liquidity of a market can be understood in terms of three facets namely; the quantity of securities that is traded (depth), the ability of the security prices to quickly recover after a liquidity shock (resiliency) and the costs incurred in trading security (tightness). Furthermore, the time taken to execute a trade (immediacy) and the intensity of trading volume impact on security prices (breadth) is also regarded as the additional facets of market liquidity [6]. (Included in the manuscript on page no. 3 in Introduction in paragraph 2) |
| 2. Moreover, adding more detailed explanation of the main results would be greatly appreciated by the reader since it provides a full picture of the main results, as well as the contribution of the study. | Included in the manuscript in Introduction on page no. 4 in paragraph 2 and on page no. 3 in paragraph 1 |
| 3. My main suggestion would be to review the abstract and introduction sections in terms of quality of written English and ensure they provide all the required information. | Included in the manuscript (Refer Abstract and Introduction) |
